# Notes and Notices

**Published:** 1889-06

**Authors:** 


### NOTES AND NOTICES.

ERRATUM.—In the May issue, page 199, second line, for *ten P. M.* read *two P. M.*

REMOVALS.—Dr. Edmund Carleton, from 58 West Ninth to 53 West Forty-fifth Street, New York city. Dr. H. W. Andrews has located at Chillicothe, Illinois. Dr. J. A. Tomhagen, from Sloan's Valley to Burnside, Kentucky. Dr. James F. Bruner, from Sedalia, Mo., to 2511 Chicago Street, Omaha, Nebraska. Mrs. M. J. Green, M. D., from Kansas City, Mo., to Chillicothe, Missouri. Dr. E. A. Smith from Uhrichsville, to South Solon, Ohio. Dr. Milton Powel from 1531 Walnut Street to 233 North Eighteenth Street, Philadelphia. Dr. W. H. Baker from 58 South Clinton Street to 77 Chestnut Street, Rochester, N. Y. Dr. J. C. White from Greenwich, Conn., to Port Chester, New Hampshire. Dr. Mary H. Baldwin from New York city to Asbury Park, New Jersey.

THE ANNALS OF SURGERY for May, 1889, has as its leading article a report by Dr. George R. Fowler, of Brooklyn, of a unique case of an air tumor of the neck caused by a hernia of the pleura in a case of pneumothorax. It is well illustrated by a lithographic plate and by a photo-engraving. The editorial articles, which are always invaluable, take up the topics of "Injuries of the Heart," "The Treatment of Cerebral Abscess," "Cancer of the Larynx," and the "Treatment of Enlarged Prostate by Electrolysis." The "Department of Index of Surgical Progress" contains an unusually copious and exhaustive series of classified abstracts of articles from foreign and domestic sources, under about forty different titles. The usual number of book reviews conclude the number. The *Annals* continues to maintain its position as a publication of the first scientific rank, one indispensable to every progressive practitioner.

FOR SALE.—Three full sets of eight volumes each of THE HOMŒOPATHIC PHYSICIAN have recently been forwarded to us for sale. Price $2.50 per volume. Single volumes will not be sold. Money must accompany the order.

Address,

THE HOMŒOPATHIC PHYSICIAN, 1123 Spruce Street.

